# Langerhans cell histiocytosis: current advances in molecular pathogenesis

**DOI:** 10.3389/fimmu.2023.1275085

**Published:** 2023-10-26

**Authors:** Tommaso Sconocchia, Johannes Foßelteder, Giuseppe Sconocchia, Andreas Reinisch

**Affiliations:** ^1^ Division of Hematology, Department of Internal Medicine, Medical University of Graz, Graz, Austria; ^2^ Institute of Translational Pharmacology, National Research Council (CNR), Rome, Italy; ^3^ Department of Blood Group Serology and Transfusion Medicine, Medical University of Graz, Graz, Austria

**Keywords:** langerhans cell histiocytosis, BRAFV600E, MAP2K1, dendritic cells, monocytes, senescence, oncogene

## Abstract

Langerhans cell histiocytosis (LCH) is a rare and clinically heterogeneous hematological disease characterized by the accumulation of mononuclear phagocytes in various tissues and organs. LCH is often characterized by activating mutations of the mitogen-activated protein kinase (MAPK) pathway with *BRAF^V600E^* being the most recurrent mutation. Although this discovery has greatly helped in understanding the disease and in developing better investigational tools, the process of malignant transformation and the cell of origin are still not fully understood. In this review, we focus on the newest updates regarding the molecular pathogenesis of LCH and novel suggested pathways with treatment potential.

## Introduction

Histiocytoses are a rare group of heterogenous hematological disorders characterized by the accumulation of histiocytes in various tissues and organs. Based on their genetic landscape and clonal features, numerous histiocystoses are now classified as myeloid neoplasms (1). The word histiocyte derives from the Greek words “*histos*” and “-cyte” which mean “tissue” and “cell” respectively. Histiocytes refer to tissue-infiltrating mononuclear phagocytes (monocytes, macrophages, dendritic cells). In the past, the classification of histiocytoses was mainly based on phenotypical features and could be divided into 3 main groups: 1) Langerhans cell histiocytosis (LCH), 2) Non-Langerhans cell histiocytosis, and 3) malignant histiocytosis. Today’s updated classification takes into account, in addition to their phenotypic features, a wide range of characteristics including molecular, pathological, and clinical features. Based on these newly included features, the new classification was divided into 5 categories: L, C, R, M, and H group. LCH and Erdheim-Chester disease (ECD) were grouped, together with mixed LCH/ECD and indeterminant histiocytosis (ICH), within the L group of the revised classification (2) (**Table 1**).

**Table 1:** Classification of histiocytic disorders.

Histiocytosis groups	
L group:	
◼ LCH ◼ ECD ◼ ICH ◼ Mixed LCH/ECD
C group:	
◼ Cutaneous non-LCH (Xanthomatous granuloma [XG] family and non XG family) ◼ Cutaneous non-LCH with a major systemic component
H group:	
◼ Primary hemophagocytic lymphohistiocytosis (HLH) ◼ Secondary HLH ◼ HLH of unknown origin
M group:	
◼ Primary malignant histiocytoses ◼ Secondary malignant histiocytoses
R group:	
◼ Familial Rosai-Dorfman Disease (RDD) ◼ Sporadic RDD (classical RDD, extranodal RDD, RDD with neoplasia or immune disease, and unclassified)

As is often the case with rare diseases, due to the scarcity of clinical samples and suitable pre-clinical models, the advancement in their understanding often proceeds at a slow rate. However, in the last 20 years, thanks to countless efforts and advancements in available technologies, numerous observations have greatly aided the understanding and management of these diseases. One major discovery was the observation that LCH cells recurrently harbor the *BRAF^V600E^* gain-of-function (GOF) mutation or other GOF mutations of the RAS/RAF/MEK/ERK cascade (3). Shortly after this seminal discovery, *BRAF^V600E^* mutations were also recurrently found in ECD patients (4). This not only led to the revisitation of the classification system but also contributed to a better diagnosis of these diseases, to the development of new pre-clinical models, and opened the way to the use of targeted therapies with BRAF and MEK inhibitors.

In this review, we will discuss the recent advances made in understanding the molecular pathogenesis of LCH. Moreover, available pre-clinical models and proposed options for targeted therapy will be discussed.

## Langerhans cell histiocytosis

**Figure 1:**
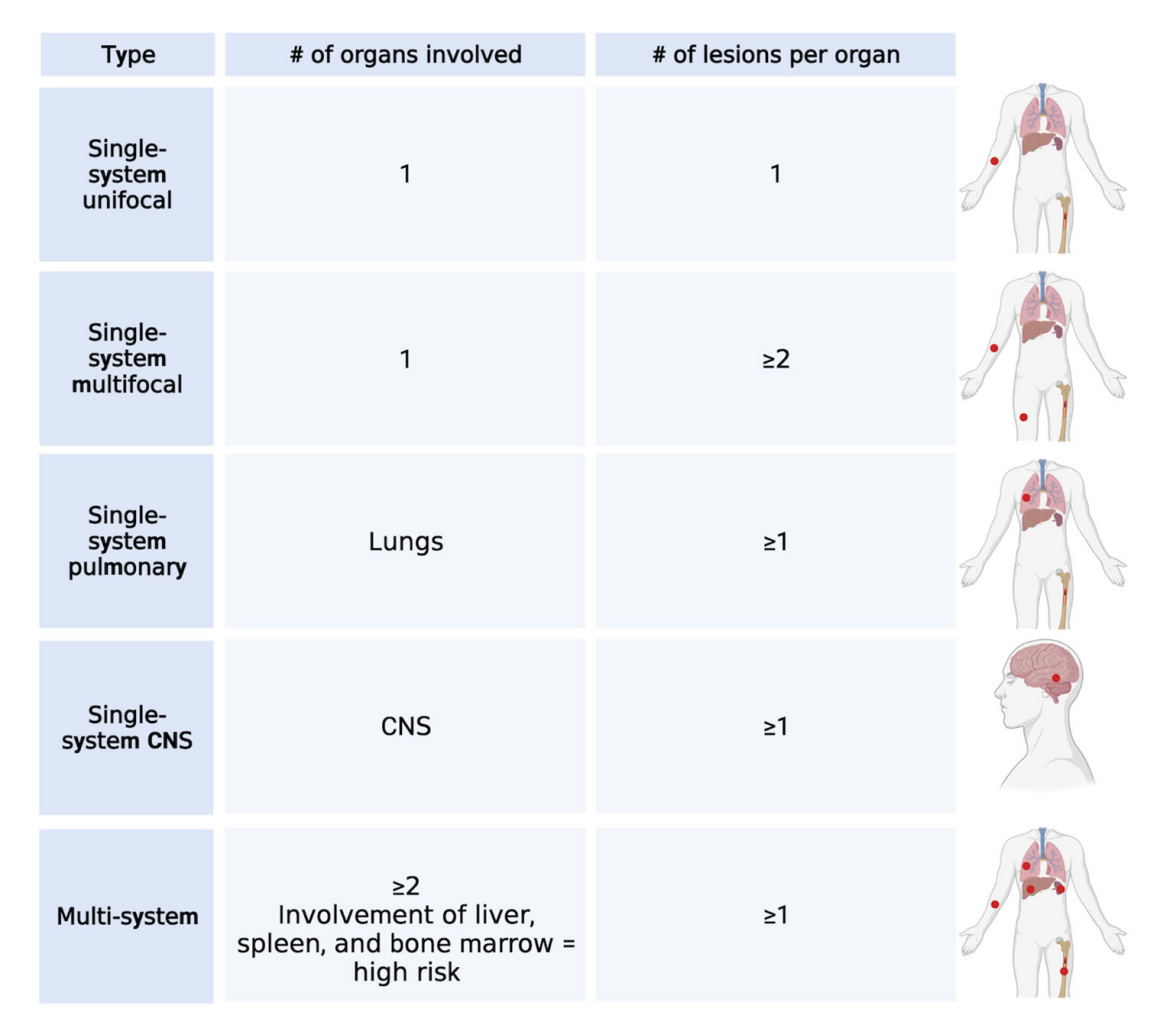
LCH classification. Main types of classification of LCH cases based on the number of lesions and number and type of organs involved. Created with Biorender.com.

LCH is a rare hematological disorder that occurs prevalently but not exclusively in children. The incidence is 3-9 cases per million children (<15 years of age) and drops to 1-2 cases per million when adults are also considered (5, 6). LCH is characterized by the infiltration of oval shaped cells with a coffee bean-like nucleus that express CD207 (Langerin), CD1a, and S100. Since almost any organ can be infiltrated by LCH cells, the clinical manifestations are very heterogenous and the type and number of organs that are involved will determine the symptoms that occur. Based on the number and type of organs and the number of lesions per organ, LCH can be classified into several single-system (unifocal, multifocal, pulmonary, and central nervous system [CNS]) and multi-system (with risk organ and without risk organ involvement) (7) subtypes (**Figure 1**). According to the classification of the Histiocyte Society, multi-system LCH is considered high risk when the liver, spleen, or bone marrow are involved (2). However, the more recent international expert consensus published in 2022 by Goyal et al. has excluded this risk stratification in the case of adult LCH because of lack of validation in adults (8). A separate classification for LCH involving only the pulmonary system has been proposed and these cases are classified as single-system pulmonary. Its separate classification derives from the fact that single-system pulmonary LCH is often associated with smoking and it is still not clear whether it is an inflammatory or a clonal proliferative disorder (9).

The bone and skin are the most common sites involved in LCH. Bone involvement is often found at the base of the skull but can also include other bones like the ribs, mandibles, vertebrae, and the long bones of arms and legs. Common manifestations are bone pain and tumor formation. When LCH involves the skin, it manifests itself mainly as seborrheic eczema (children) and refractory eczema (adults). When only the bone or skin are involved (unifocal, single-system, or multi-system without the involvement of risk organs), patients usually have a good prognosis and either require minimal treatment or the lesions regress spontaneously. With appropriate treatment, the overall survival of patients is 100% but up to half of the survivors will present lasting consequences. In the case of disseminated multi-system LCH with risk organ involvement, the survival rate drops to 70% and reactivation of the disease is observed in around one-third of the patients once the treatment is completed. Therefore, even though much progress has been made in the treatment of LCH, it is important to get a deeper understanding of this disease and to identify alternative treatment strategies.

## Mutational landscape

**Figure 2:**
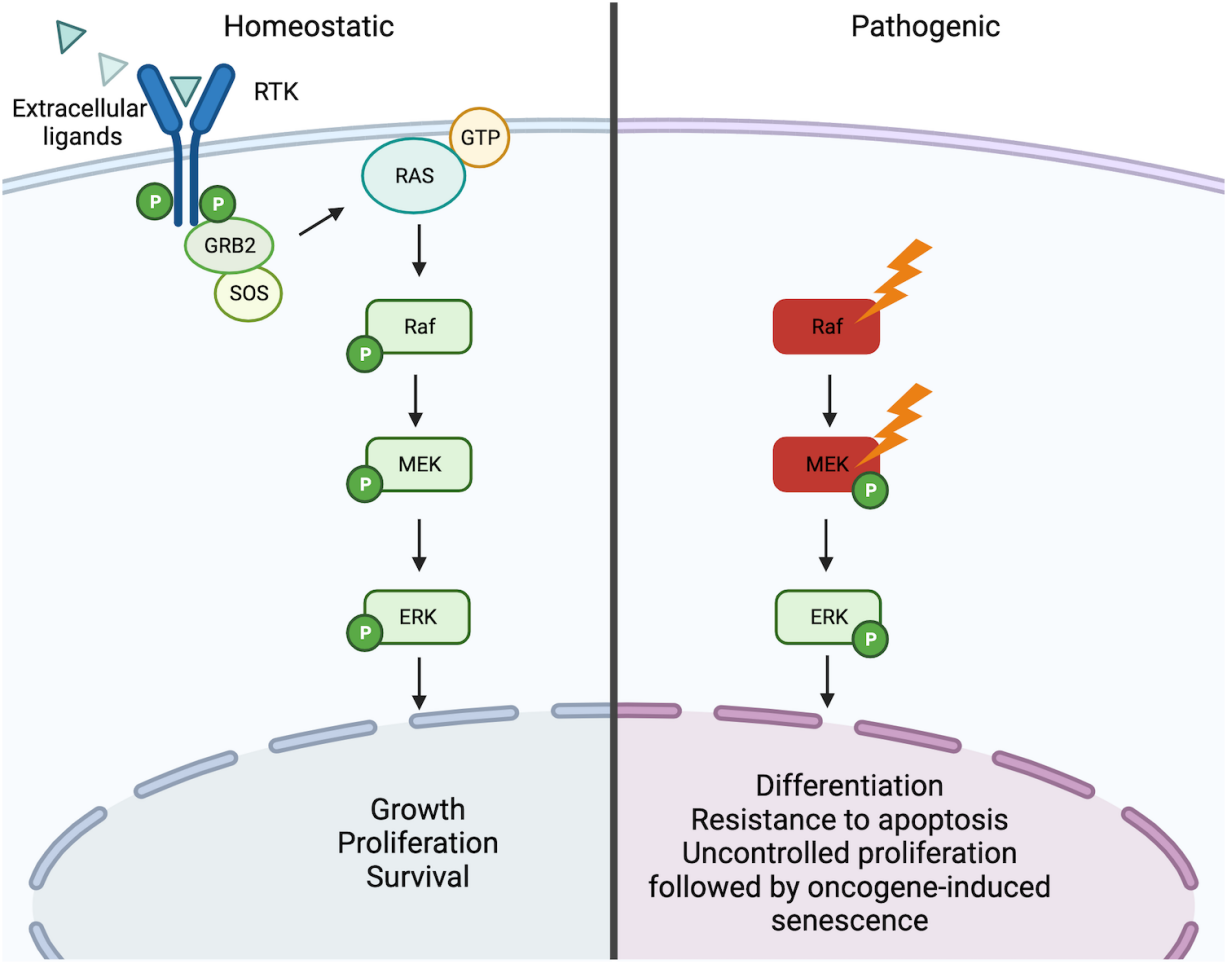
RAS/RAF/MEK/ERK signaling pathway. Under homeostatic conditions the RAS/RAF/MEK/ERK is activated through the binding of extracellular ligands (i.e. hormones, growth factors, and cytokines) to receptor tyrosine kinases (RTKs) leading to dimerization and phosphorylation. In turn, this leads to the induction of the active form RAS-GTP, which leads to a downstream cascade that is characterized by the activation through phosphorylation of Raf, MEK, and ERK. Activated ERK can then trigger the transcription of numerous genes involved in the growth, proliferation, and survival of the cell. In the presence of activating mutations of Raf (i.e. *BRAF^V600E^*) or MEK (MAP2K1 mutations), this pathway becomes constitutively active independent of the ligand-receptor interaction. Constitutive activation of the pathway can consequently lead to oncogenesis through induced cell differentiation, resistance to apoptosis, and oncogene-induced senescence. Created with Biorender.com.

The most frequently recurrent mutation in LCH is the *BRAF^V600E^* mutation (3). The v-Raf murine sarcoma viral oncogene homolog B (*BRAF*) is a gene encoding for the homonymous BRAF protein, a serine/threonine-protein kinase, that together with its homologs ARAF and CRAF, belongs to the mitogen-activated protein kinase (MAPK) signaling pathway. This pathway governs numerous processes which influence cell growth, proliferation, differentiation, and survival (10–12). The signaling cascade begins with an activator signal (i.e. hormones, growth factors, and cytokines) followed by a sequential activation of MAPKs (MAPKKK, MAPKK, MAPK) and each component is activated by the upstream MAPK through autophosphorylation of their serine-threonine residues. Currently, 4 MAPK pathways have been described and these include the p38 MAPK, the extracellular-signal regulated (ERK) 1/2, ERK5, and c-Jun N-terminal kinase (JNK). BRAF is a core component of the MAPK/ERK1/2 signaling cascade and involves the sequential phosphorylation and activation of RAS-RAF-MEK-ERK (13) (**Figure 2**).

The *BRAF^V600E^* mutation is a GOF mutation that consists in the substitution of a thymine (T) with an adenine (A) in exon 15 (position 1799) of the BRAF gene. This single base substitution leads to the replacement of the amino acid valine (V) in position 600 with a glutamate (E) leading to constitutive activation of the BRAF protein which subsequently results in the constitutive activation of the downstream components MEK and ERK1/2 (14). *BRAF^V600E^* was first observed in LCH by Badalian-Very et al. in 2010. In this study, DNA was extracted from paraffin blocks containing LCH biopsies from different lesion sites. Mass spectrometry and pyrosequencing detected *BRAF^V600E^* mutations in ∼57% of the cases (3). This discovery was later confirmed by several other studies (15–18).

The remaining cases of LCH that do not bear the *BRAF^V600E^* mutation are often characterized by other mutations in the *BRAF* gene, mutations in *ARAF*, other activating mutations in the MAPK pathway downstream of *BRAF* (**Tables 2**, **3**), and other activating mutations (i.e. *CSF1R (* 17), *ERBB3 (* 18)). Among the non-*BRAF^V600E^* mutations, the activating *MAP2K1*, encoding for MEK, is the most prevalent mutation with an incidence of ∼ 30% (16, 17, 19–25).

**Table 2:** RAF mutations identified in Langerhans cell histiocytosis.

Gene	AA Mutation	Type	References
*BRAF*	p.V600E	Substitution	(3, 17, 19)
	p.R506_K506insLLR	Splicing	(20)
	p.V600insDLAT	Insertion	(21)
	p.V600D	Substitution	(17, 22)
	p.N486_P490del	Deletion	(17, 23)
	BICD2-BRAF	Fusion	(17)
	p.R603Q	Substitution	(17)
	PACSIN2-BRAF	Fusion	(17)
	SPPL2A-BRAF	Fusion	(17)
	p.H353R	Substitution	(17)
	p.X504_splice	Splicing	(17)
	FAM73A-BRAF	Fusion	(23)
	p.485_490LNVTAP>F	Substitution	(23)
	p.486_491NVTAPT>K	Substitution	(23)
*ARAF*	p.V263M	Substitution	(17)
	p.F351L	Substitution	(24)
	p.Q347_A348del	Deletion	(24)

**Table 3:** Non-*RAF* mutations identified in Langerhans cell histiocytosis.

Gene	AA Mutation	Type	References
KRAS*	p.G12S	Substitution	(17)
NRAS*	p.G12D	Substitution	(17)
*MAP2K1*	p.Q56P	Substitution	(16)
	p.Q58_E62del	Deletion	(16, 17)
	p.F53_Q58delinsL	Deletion/Insertion	(16, 17, 19)
	p.E102_I103del	Deletion	(16, 17, 19)
	p.R47Q	Substitution	(19)
	p.K57G61del	Deletion	(19)
	p.I99_R104del	Deletion	(19)
	p.H100_I103delinsPL	Deletion/Insertion	(19)
	p.R49C	Substitution	(19)
	p.A106T	Substitution	(19)
	p.C121S	Substitution	(19)
	p.G128V	Substitution	(19)
	p.K57T	Substitution	(17)
*MAP3K*	p.V121M	Substitution	(17)
	p.E1286V	Substitution	(18)
	p.T779fs	Frame-shift	(18)
	p.T1481fs	Frame-shift	(18)
*MAPK7*	p.R400L	Substitution	(17)
MAP2K6*	p.E102_I103delEI	Deletion	(17)
MAP3K4*	p.M1415I	Substitution	(17)
MAPK11*	p.D227Y	Substitution	(17)
MAP3K9*	p.R303L	Substitution	(17)

The asterisk (*) indicates that the mutations are co-occurring with other mutations.

## Cell of origin

**Figure 3:**
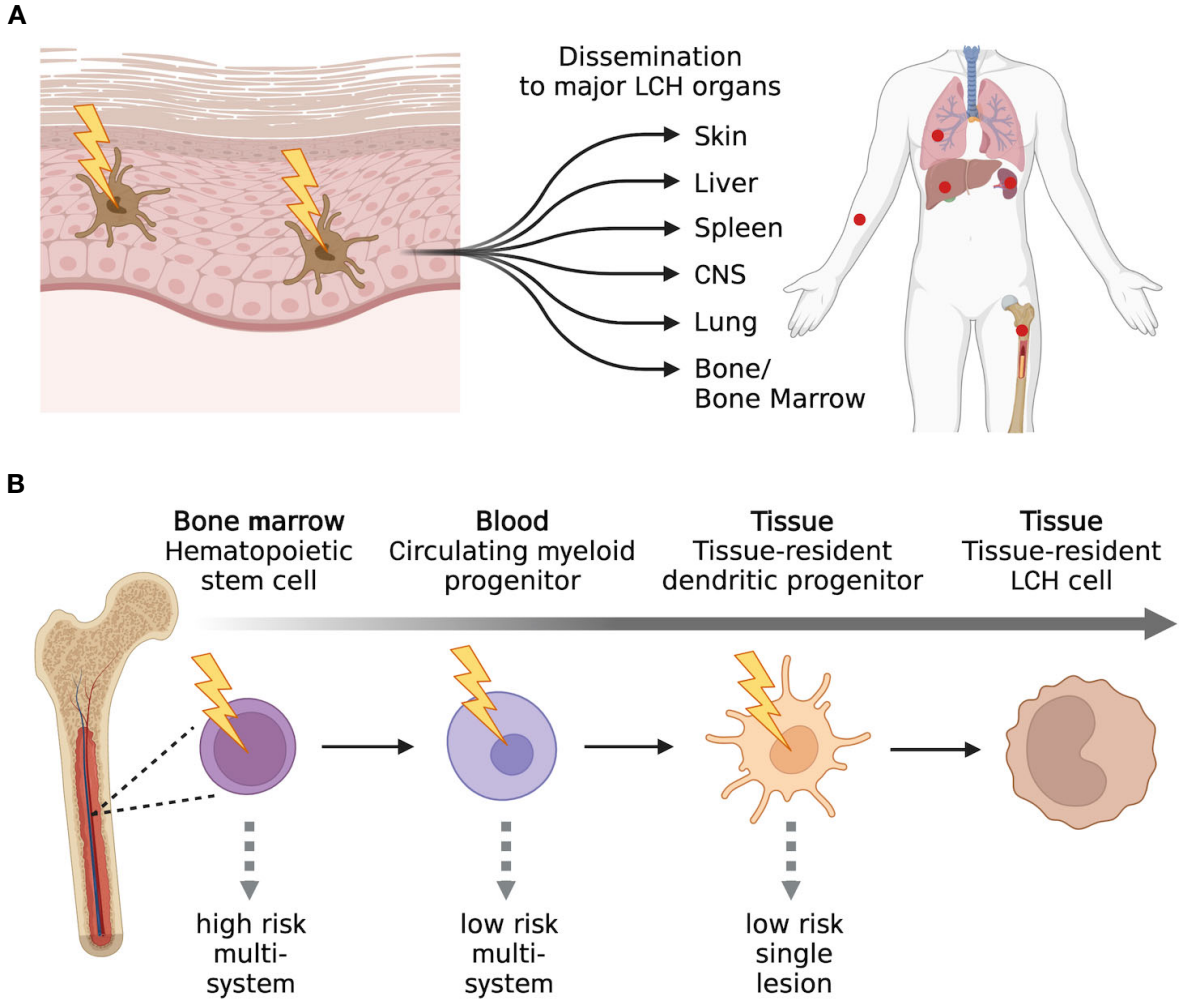
Past and present models of LCH. **(A)** The “Activated-Immature” model of LCH was one of the first models proposed. In this model, it was proposed that LCH was a consequence of the malignant transformation of LCs present in the dermis. Through this malignant transformation, the aberrant LCs could proliferate, migrate, and form lesions in numerous compartments of the human body. The “Activated-Immature” model of LCH was then replaced by **(B)** the “Misguided Myeloid DC precursor” model. This model suggests that LCH cells can arise from different myeloid precursors and the stage of differentiation of the precursor cells will determine the severity of the disease. Created with Biorender.com.

LCH cases were first unified under a common name known as Histiocytosis X. This was proposed by Lichtenstein in 1953 and allowed to group the previously described cases that were variations in the localization, involvement, and degree of the same disease. Its purpose was to simplify and replace the old and more confusing nomenclatures. The decision to rename this disease as histiocytosis X derived from its unknown etiology and from the fact that the infiltration of proliferating cells in various tissues and organs was the common feature of the different forms of this disease. The subsequent observation in 1973 by Nezelof et al., that the infiltrating cells in the lesions contained Birbeck granules was a turning point in trying to identify the cell of origin of LCH (26). Birbeck granules are “tennis-racket”-shaped organelles located in the cytoplasm that are almost unique to Langerhans cells (LCs) located in the skin (27). This observation allowed to first link histiocytes to LCs and subsequently led to the replacement of the term “Histiocytosis X” with “Langerhans cell histiocytosis”. The close phenotypical resemblance of LCH cells with epidermal LCs (CD1a, CD207, and Birbeck granule positivity) (28–31) led researchers to hypothesize that the cell of origin of LCH were pathological epidermal LCs and to propose the “Activated-Immature Model” (32) (**Figure 3A**).

The “Activated-Immature Model”, in which pathological activated-immature LCs are considered the initiating cells of LCH and disseminate into various organs, was later surpassed and replaced by the “Misguided Myeloid Dendritic Cell Precursor” model (**Figure 3B**). The latter model suggests that LCH cells arise from aberrant myeloid progenitors that differentiate into CD1a^+^CD207^+^ LCH cells following migration into the LCH sites. Supporting this model was the observation that the gene expression profile of CD207^+^ LCH cells isolated from patients was distinct from healthy epidermal LCs that were used as controls. In addition, CD207^+^ LCH cells were enriched in genes encoding for early myeloid markers (33). Another observation that moved the scientific community from the “Activated-Immature Model” to the “Misguided Myeloid Dendritic Cell Precursor” model was the fact that CD207, which was previously thought to be a unique LC marker, can also be expressed in other DC subsets (34–36). Further support stems from the fact that LCs can be differentiated *in vitro* from hematopoietic CD34^+^ progenitors, CD14^+^ monocytes, and CD1c^+^ DCs in the presence of members of the TGFβ superfamily (i.e. TGF-β1, BMP7) (37–43). In order to efficiently differentiate into LCs, CD14^+^ monocytes require Notch ligation in addition to TGFβ (44). Interestingly, active Notch signaling can be detected in LCH lesions, and the Notch agonist Jagged-2 (Jag2) was described to promote the differentiation of CD14^+^ monocytes into CD1a^+^CD207^+^ LCs that express a gene-signature similar to that of LCH cells (45, 46). On the other hand, CD1c^+^ DCs can readily differentiate into LCs in the presence of TGFβ and do not require Notch ligation (43). In addition, transcriptional profiling performed by Lim et al., revealed that the transcriptional profile of circulating CD1c^+^ DCs was the most closely related to that of CD1a^+^CD207^+^ LCH cells when compared with the profiles of the other myeloid subsets (47).

The discovery that genes of the MAPK pathway are recurrently mutated in LCH allowed to better and more easily perform studies regarding the cell of origin of LCH. Initial studies tracing the *BRAF^V600E^* mutation in hematopoietic populations sorted from adult LCH patients revealed interesting results. In fact, the *BRAF^V600E^* mutation was not only detected in the previously described myeloid populations that are able to differentiate into CD1a^+^CD207^+^ LCs but also in CD16^+^ monocytes, CD123^+^ plasmacytoid DCs (pDCs), T cells, and B cells. Moreover, the mutation was also detected in hematopoietic stem and progenitor cells (HSPCs) and this provided first evidence of a hematopoietic stem cell origin of LCH (48, 49). In addition, a strong correlation between the differentiation stage of the cell bearing the mutation and disease severity could be determined, with the higher risk disease being associated with the presence of the mutation in the HSPC compartment (**Figure 3B**). An HSPC origin of LCH was additionally supported by xenograft studies. Transplantation of CD34^+^ HSPCs engineered to express the *BRAF^V600E^* or *MAPK2K1* mutation into immune deficient mice led to the onset of LCH-like disease (50–52).

A more recent study that additionally included recurrent non-*BRAF^V600E^* mutations revealed a similar pattern to the *BRAF^V600E^* studies. In addition, this study, by separating the CD1c^+^ DCs into DC2s and recently described DC3s (53), was able to show that MAPK pathway mutations are present in both subsets (54) suggesting that both CD1c expressing DC subsets can be precursors to LCH cells. DC3s, similar to monocytes and in contrast with DC2s, require Notch signaling to differentiate into CD1a^+^CD207^+^ cells (55).

Despite all the recent advances in understanding the origin of LCH, the precise cell of origin is still not clearly defined. The most recent theory accepted by the scientific community is an updated version of the “Misguided Myeloid Dendritic Cell Precursor” model in which more than 1 cellular subset can give rise to LCH.

## Molecular pathogenesis

### Cell intrinsic effects

To better understand cell intrinsic abnormalities involved in the pathophysiology of LCH, studies are increasingly focusing on the use of rare LCH biopsy specimens. An alternative approach to elucidate cell-intrinsic effects represents *in vitro* and *in vivo* mutation modeling that relies on the insertion of the *BRAF^V600E^* or *MAP2K1* mutations.

Since it was initially suggested that LCH cells are abnormal epidermal LCs, the first studies focused on comparing phenotypic differences between LCH cells and LCs. It was soon discovered that although LCH cells express the hallmark LC markers CD1a, CD207, and Birbeck granules, LCH cells are characterized by numerous peculiarities. LCH cells do not exhibit the characteristic dendritic cell-like protrusions but are round/oval in shape and have a coffee-bean like nucleus (56). Immunophenotyping performed on biopsies from LCH patients revealed the co-expression of CD1a, CD207, S100, CD36, CD40, and the monocytic/macrophage markers CD14 and CD68. Additionally, LCH cells were shown to rarely express CD83 and DC-LAMP (CD208) (15, 57). Some LCH cells were detected to express CD80 and CD86 suggesting that LCH cells can be found in different maturation stages (15, 57, 58). E-Cadherin (CD324), a marker that is strongly expressed by LCs and mediates their adhesion to keratinocytes (59), is either absent or expressed at very low levels and its absence was suggested to be associated with poor prognosis (33, 60). LCH cells were also described to express lower levels of the major histocompatibility complex (MHC) class 1 genes (55), high levels of programmed cell death 1 ligand 1 (PD-L1; CD274) (61), PD-L2 (CD273) (62), receptor activator of nuclear factor kappa-B ligand (RANKL; CD254) (63), and inducible costimulatory factor ligand (ICOSL; CD275) (64) contributing to immune escape by LCH cells in the lesions.

LCH cells were also shown to be characterized by high signal regulatory protein α (SIRPα) expression (65). SIRPα is a protein that is normally expressed on the surface of immune cells with phagocytic capacity like monocytes, DCs, and macrophages. It acts as a receptor to CD47 and performs an innate immune checkpoint function. The binding of CD47 to SIRPα constitutes a “don’t eat me” signal masquerading the cell from the phagocytic activity of macrophages (66). Treatment of transgenic mice specifically expressing *BRAF^V600E^* on DCs (BRAFV600E^CD11c^ mice) with an anti- SIRPα antibody was able to decrease the severity of disease (65). However, the exact mechanism still remains to be further elucidated.

LCH cells were described to be the only cells in lesions that co-express Notch ligands and Notch receptors. Immunofluorescence analysis of lesions determined that CD207^+^ LCH cells exhibited active intracellular Notch signaling suggesting that LCH cells interact with each other activating this pathway (45). Moreover, a cooperativity between two LCH populations, one having a DC2 signature and the other a DC3/monocyte signature, was implicated. It was suggested that the DC2-like LCH cells could stimulate the DC3/monocyte-like LCH cells to acquire the CD1a^+^CD207^+^ phenotype by activating the Notch pathway in the latter population (55). Therefore, an important role in LCH development and function was attributed to the Notch signaling pathway and targeting this pathway, for example through the use of gamma-secretase inhibitors, could represent an interesting therapeutic option for further investigations.

Bulk and single-cell analysis of LCH lesions led to a better characterization of the cell intrinsic abnormalities in LCH cells. Bulk analysis of the transcriptome of LCH cells could confirm that LCH cells are more closely related to myeloid progenitors and DCs rather than epidermal LCs (33, 47, 67). These analyses identified numerous interesting differentially expressed genes in LCH. Among these, LCH cells are characterized by high expression of genes encoding for the anti-apoptotic proteins of the BCL2 family (*BAX* and *BCL2L1*) *(* 33). The increase in the anti-apoptotic genes was a consequence of the constitutive activation of the MAPK pathway in the cells (68).

Subsequent single-cell RNA sequencing (RNAseq) studies further elucidated the complex aberrations of LCH cells. Halbritter et al. observed a developmental hierarchy in LCH cells and could identify 14 different subsets. The subsets ranged from having an undifferentiated cell signature with proliferative properties (*MKI67*, *AURKA*, *AURKB*), resembling proliferative progenitor-like LCH cells, to a more differentiated signature with highly expressed genes associated with dendritic cells and inflammation (*BATF3*, *IRF8*, *MMP9*, *MMP12*), resembling more mature LCH cells (69). A more recent study by Shi et al. confirmed the heterogeneity of LCH cells in skin lesions by identifying, through single cell RNAseq, 4 major subsets with 1 subset expressing numerous genes related to proliferation (*MKI67*, *CENPF*) *(* 70).

### Oncogene-induced senescence

A recent discovery that strongly changed our understanding of LCH was the observation that the constitutive activation of the MAPK pathway by the *BRAF^V600E^* mutation induced a senescent program in LCH (51, 52, 55). It was in fact shown that overexpression of *BRAF^V600E^* in CD34^+^ HSPCs led to striking changes in the cell’s gene function and gene expression. When compared to healthy HSPCs, *BRAF^V600E^* HSPCs were characterized by an initial proliferative advantage followed by a proliferative disadvantage in later stages of differentiation. Moreover, HSPCs bearing the MAPK pathway activating mutation had reduced clonogenic properties and were biased towards myeloid lineage, differentiating at the expense of the lymphoid lineage (52). We could confirm these observations and also demonstrate that the effects are not simply due to the overexpression of the mutation by utilizing a novel CRISPR/Cas9-based system (71) to mono-allelically insert the *BRAF^V600E^* mutation in the endogenous locus of the BRAF gene of healthy human HSPCs and therefore, maintain its endogenous expression (72).

In terms of their transcriptome, enforced expression of *BRAF^V600E^* increased the expression of genes associated with cellular senescence. These included: *CDKN2A*, *CDKN2C*, *CDKN2D*, and *CDKN1A* (genes encoding for the cell cycle regulators p16^INK4A^, p18^INK4C^, p19^INK4D^, and p21^CIP1^), *CD9*, and *MDM2*. Moreover, high expression levels were detected for matrix metalloproteinases (MMPs), *IL6*, *IL8*, *MCP1*, *TNFA*, *IL1A*, and *IL1B* (52, 72). These factors are known to be associated with a senescence-associated secretory phenotype (SASP). Although initially capable of promoting the clearance of cancer cells by recruitment of immune cells to LCH lesion sites, chronic SASP with sustained secretion of pro-inflammatory cytokines and chemokines can have opposing effects and eventually lead to immune escape and cancer progression (73).

Immunohistochemistry and RNAseq of LCH biopsies confirmed the presence of senescent cells in LCH. LCH cells from lesions showed absent or very low expression of the proliferation marker Ki67, whereas numerous cells stained positive for senescence-associated beta-galactosidase (SAβGal), and p16INK4a (51). In addition, the gene signature of sorted LCH cells was comparable with the gene signature of *in vitro* generated *BRAF^V600E^* mutant cells with high expression of the above mentioned genes encoding for negative cell cycle regulators and SASP factors (51, 55).

Targeting senescent cells may represent a valid therapeutic option for treating LCH. Various diseases are linked to the accumulation of senescent cells and pre-clinical models that target the senescent cells have reported alleviation of disease symptoms (74). Regarding LCH, two recent studies have addressed this strategy confirming beneficial effects in regards to disease progression (51, 52).

### Microenvironment

**Figure 4:**
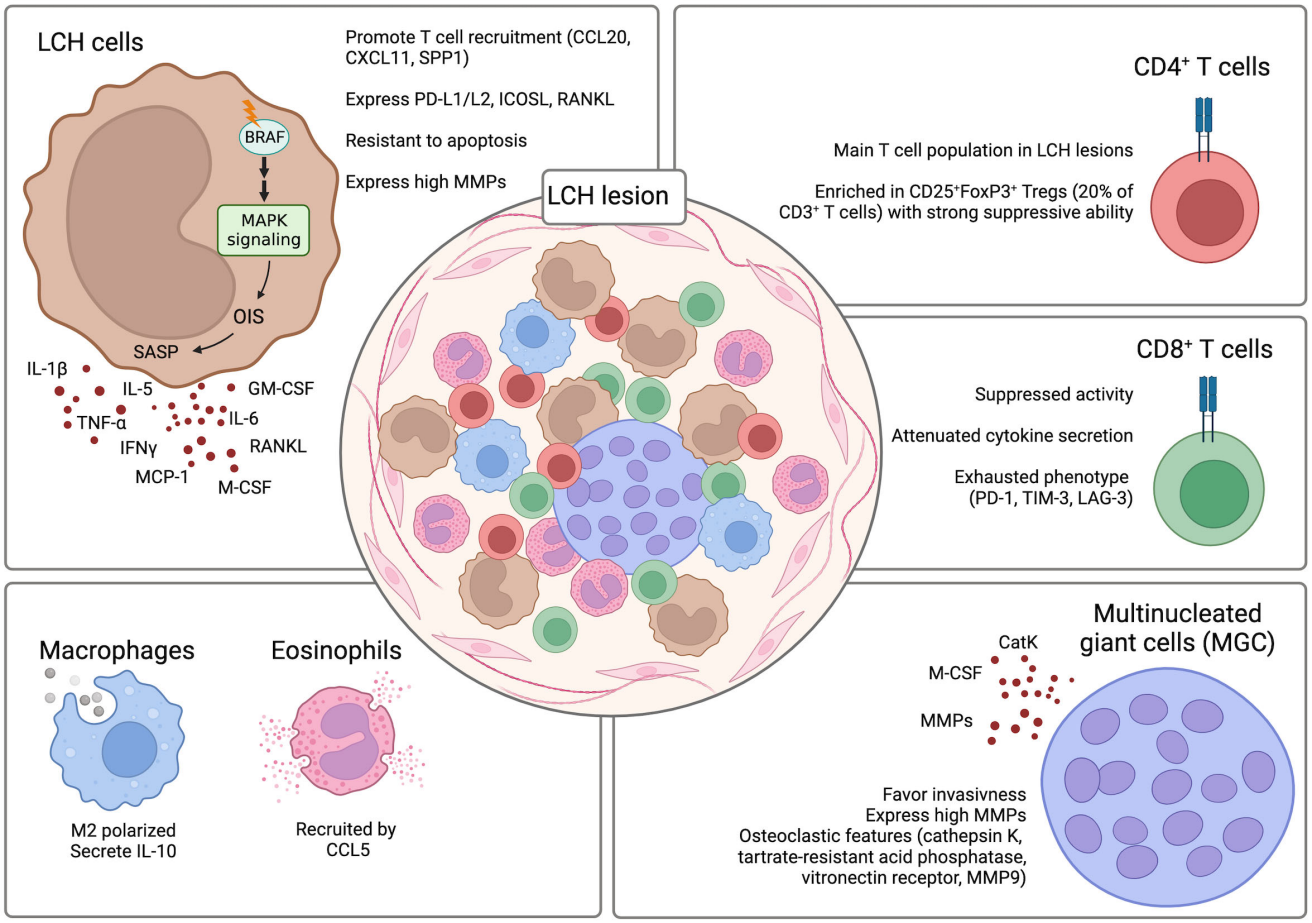
LCH lesion composition. The composition of LCH lesions is very heterogenous with the presence of different components of the immune system. Crosstalk between the LCH cells and the infiltrating immune cells contributes to the formation of a highly inflammatory and dysfunctional microenvironment that inhibits the clearance of the LCH cells by the immune cells and favors their persistence and survival in the lesions. Created with Biorender.com.

The composition of LCH lesions is very heterogeneous and does not only include LCH cells. In fact, the lesions are populated by varying amounts of LCH cells that differ between patients and can range from less than 10% to more than 50% of LCH cells (47, 75, 76). In the case of *BRAF^V600E^* positive LCH, given that LCH is a clonal disorder, it was shown that CD1a^+^CD207^+^ LCH cells within the lesions are almost in their entirety harboring the mutation (47). The remaining population is comprised of other immune cells including T cells, myeloid-derived suppressor cells (MDSCs), macrophages, pDCs, eosinophils, B cells, and multinucleated giant cells (MGCs) (55, 69, 77). A constant and reciprocal interaction between LCH cells and the other infiltrating immune cells was suggested to contribute to the pathogenesis of the disease and to the establishment of a highly inflammatory microenvironment. The presence of such an inflammatory environment has given the rationale for the combinatorial use of corticosteroids with chemotherapeutic compounds as first line treatment. Interestingly, although LCH lesions are characterized by this intense inflammatory setting, paradoxically numerous immunosuppressive elements can also be detected within the lesions (i.e. TGF-β1, IL-10, CD25^+^FoxP3^+^ regulatory T [Treg] cells). This creates a microenvironment that favors the persistence of LCH cells in the lesions and inhibits their clearance (**Figure 4**).

Among the immune cells present in LCH lesions, pDCs could be a useful biomarker for determining disease severity in LCH, since lower frequencies of pDCs were associated with a more severe disease (70).

T cells, together with LCH cells, represent one of the largest populations in LCH lesions and have been suggested to play a role in the pathogenesis of LCH. LCH cells can produce various factors like CCL20, CXCL11, and SPP1 that can recruit T cells to the lesions (33, 78). CyTOF analysis of LCH lesions could determine that CD4^+^ T cells are present at significantly higher percentages as compared to the CD8^+^ T cell compartment (74% +/- 12.26% vs. 21% +/- 5.61%) (62). Among the CD4^+^ T cells, CD25^+^FoxP3^+^ Treg cells were particularly enriched and constituted around 20% of the total CD3^+^ T cells (63, 79). Importantly, the Tregs are not dysfunctional and retain the ability to suppress (80). A direct role of LCH cells in inducing the differentiation of CD25^+^FoxP3^+^ Treg cells was suggested. LCH cells were implicated in promoting the expansion of CD25^+^FoxP3^+^ Treg cells due to their high expression of PD-L1, PD-L2, ICOSL, and RANKL which are factors previously described to play a role in the differentiation of CD25^+^FoxP3^+^ Treg cells (57, 81–84). Whereas the CD25^+^FoxP3^+^ Treg cells were described to be functional and maintain their suppressive ability, CD8^+^ T cells present in the LCH lesions are mostly dysfunctional. Infiltrating CD8^+^ T cells are characterized by an attenuated alloreactivity and a reduced ability in secreting cytokines even after stimulation. Moreover, they were described to have an exhausted phenotype with increased expression of co-inhibitory molecules PD-1, TIM-3, and LAG-3 (62). In addition to conventional T cell lineages, also unconventional T cell subsets have been investigated in LCH patients. Among the analyzed subsets, a decrease of mucosal-associated invariant T (MAIT) cells in the lesions and peripheral blood of LCH patients was observed (85). However, whether they have a role in the pathogenesis of LCH is still not known.

The role of other immune cell populations that infiltrate LCH lesions has not yet been clearly elucidated. However, they have also been implicated in LCH pathogenesis. Macrophages were detected to be differentiated towards the M2 phenotype and to be positive for IL-10 expression thus favoring persistence of LCH cells by inhibiting their clearance by the other immune cells populating the lesions (86). Although eosinophils are an important infiltrating population of LCH lesions, their exact role is not yet known. It was suggested that their recruitment is mediated by CCL5 expression by LCH cells (78).

An interesting finding was the detection of MGCs in LCH lesions (76). It is hypothesized that MGCs, through the expression of MMPs, can have a destructive role and favor invasiveness in the lesions (77). MGCs express osteoclastic features (cathepsin K, tartrate-resistant acid phosphatase, vitronectin receptor, MMP9) with expression of monocytic/macrophage markers (i.e. CD68) (77). Interestingly, the MGCs in non-osseous lesions co-express CD1a suggesting that they may be differentiated *in situ* rather than recruited to the lesions (77). This may be explained by the high presence of osteoclast-promoting factors like osteopontin, IL-1, IL-6, TNFα, IL-17, and colony-stimulating factor 1 (CSF1) (87–89). The high presence of osteoclast-promoting factors can induce the differentiation of osteoclast-like MGCs from precursors like immature DCs and monocyte/macrophages (90, 91).

Regarding the role of IL-17A, there is some controversy. IL-17A is a cytokine that is mainly produced by lymphoid cells (i.e. CD4 and CD8 T cells, innate lymphoid cells, gamma-delta T cells, and NKT cells) (92). Under some circumstances, IL-17A is also produced by myeloid cells, although this process is not as clear (93–96). High levels of IL-17A can be found in different inflammatory diseases and since LCH shares numerous symptoms with that of IL-17A-driven disease, it was hypothesized that it could also play an important role in LCH pathogenesis. Coury and colleagues could show that active LCH was characterized by high serum levels of IL-17A and that quite unexpectedly one of the main sources of IL-17A were LCH cells (97). They additionally demonstrated that IL-17A was able to promote DC fusion and the generation of small MGC that expressed acidic phosphatase, MMP9 and MMP12 (97). However, this observation promoted some debate in the scientific community (98, 99), as later studies failed to detect the expression of IL-17A in LCH cells both at the mRNA and protein level (100, 101). A follow-up study tried to settle this debate by analyzing the expression of the receptor for IL-17A (IL-17RA). Comparing multi-system LCH to single-system LCH, this study demonstrates that multi-system LCH has higher IL-17RA expression. It was also established that serum levels of IL-17A are elevated in LCH, but IL-17A is not expressed by LCH cells (102).

High CSF1 expression in LCH lesions was also suggested to contribute to LCH pathogenesis. It was shown that both, ligand (CSF1) and receptor (CSF1R) are expressed in LCH lesions and that CSF1 can promote CD1a^+^CD207^+^ LC differentiation (103). This suggests that the high expression of this growth factor may have an important role in the differentiation or recruitment of LCH cells to the lesions and that targeting CSF1R with inhibitors (i.e. BLZ945) could have a beneficial therapeutic effect. However, since differentiation and inhibition experiments were performed only *in vitro* using healthy monocytes, CD34^+^ HSPCs, and CD1c^+^ DCs (103), additional *in vitro* (i.e. *BRAF^V600E^* engineered HSPCs) and *in vivo* (i.e. *BRAF^V600E^* transgenic or humanized mice) experiments are necessary to validate this claim.

## Newly proposed directions in the therapeutic treatment of LCH

The development of efficient therapies for LCH has seen some difficulties throughout the years. This is mainly due to the very heterogenous forms in which LCH can manifest itself and the lack of knowledge of the pathobiology of the disease. Therapy strongly depends on where and how many sites are involved. The majority of cases with single-system LCH are curable. Lesions involving the bone can be treated with curettage, intralesional corticosteroid therapy, radiotherapy, bisphosphonates, oral methotrexate (MTX), or hydroxyurea (HU). Depending on the severeness of the skin lesions and the physician and/or patient preferences, skin lesions can be treated with either local or systemic therapy. Topical treatment (i.e. triamcinolone) is used in the case of small localized skin lesions, whereas in case of more extensive lesions therapies vary and can include HU, MTX, 6-mercaptopurine (6-MP), vinblastine/vincristine, cytarabine (AraC), cladribine (2-chloro-2’-deoxyadenosine [2-CdA]), and/or thalidomide (104). For patients with multisystem LCH, systemic therapy should be considered. Based on the effectiveness of nucleoside analogues in the treatment of myeloid malignancies, AraC, 2-CdA, and clofarabine have shown effectiveness also in the treatment of LCH (105–107).

First line therapy consisted in treatment with vinblastine and prednisone. Patients that did not respond to two courses of first line treatment were recommended for second-line treatment consisting in Ara-C and 2-CdA. Clinical trials organized by the Histiocyte Society have aimed at improving the treatment conditions of patients with multisystem LCH. The LCH-II trial (ISRCTN57679341) provided evidence that therapy intensification by addition of etoposide to prednisone, vinblastine, and 6-MP reduced the mortality in patients with multisystem LCH involving risk organs (RO+) (108). However, the reactivation rate for these patients was still high. The LCH-III study (NCT00276757) was then initiated to further improve the treatment of multisystem LCH. Although replacing etoposide by MTX did not provide the envisioned benefit, the study could show that patients benefited from a prolongation of the therapy from 6 to 12 months (109). Currently, the LCH-IV study (NCT02205762) is underway and aims at better tailoring the therapy to the clinical presentations of the disease by further extending the therapy duration from 12 to 24 months depending on low or high risk.

The observation of recurrent MAPK mutations in LCH has provided a very strong rationale for a targeted therapy that aims at inhibiting the activated components of the pathway (i.e. RAF and MEK inhibitors). Early-phase clinical trials and case reports have reported that MAPK pathway inhibitors are effective with high response rates (67, 110–114). Although the treatment with inhibitors of the MAPK pathway resulted in a quick improvement of symptoms and control of the disease, they have not yet proven to be curative when used alone. Drawbacks of therapies with MAPK pathway inhibitors are high relapse rates following discontinuation of the therapy and a toxicity profile which still needs to be further defined (111, 112, 115–117). Currently, various clinical trials are either recruiting or active in order to determine the optimal therapeutic conditions for MAPK inhibitors and whether current treatment strategies can benefit from the combination with MAPK inhibitors (**Table 4**).

**Table 4:** Active and recruiting clinical trials investigating inhibitors of the MAPK pathway for the treatment of Langerhans cell histiocytosis.

NCT number	Description	Status
NCT05828069,	Phase II trial evaluating tovorafenib (DAY101) in patients with progressive, relapsed after treatment, and refractory LCH.	Recruiting
NCT05092815,	Phase II trial investigating the BRAFV600E inhibitor HLX208 in patients with LCH and Erdheim-Chester disease.	Active, not recruiting
NCT04079179,	Phase II trial investigating the MEK inhibitor cobimetinib in patients with various histiocytic disorders including LCH.	Recruiting
NCT03585686,	Phase II trial investigating the combination of the BRAFV600E inhibitor vemurafenib with low dose Ara-C and 2-Cda.	Recruiting
NCT03220035	Phase II trial investigating vemurafenib in pediatric patients with advanced, recurrent or refractory LCH.	Active, not recruiting
NCT04943224	Phase II trial aiming to optimize the dose and timing of trametinib in patients with BRAF negative refractory LCH or after failure with vemurafenib.	Recruiting
NCT03698994	Phase II trial investigating the ERK1/2 inhibitor ulixertinib (BVD-523FB) in patients with tumors with MAPK mutations including LCH.	Active, not recruiting
NCT04943198	Phase II trial aiming to optimize the dose and timing of vemurafenib in patients with BRAF positive refractory histiocytosis.	Recruiting

Beyond the introduction of MAPK pathway inhibitors in the treatment of LCH, other potential therapies are starting to be proposed and investigated. Given the importance of AKT signaling for the survival of DCs and its upregulation in hematological diseases, a phase II clinical trial investigated the efficacy of the pan-AKT inhibitor afuresertib on a cohort of patients with *BRAF^V600E^* and wild-type LCH. However, since the overall response rate was only 30% it was not deemed to be interesting for further development (118).

Immunotherapy is another promising strategy currently being investigated for the treatment of LCH. A phase I trial for investigating the use of CD207-CAR-T cells for the treatment of relapsed and refractory LCH is active but patients are not yet being recruited (NCT05477446). Another suggested immunotherapeutic concept is the combined use of PD-1/PD-L1 and MAPK pathway inhibitors. This concept is based on interesting pre-clinical data demonstrating that the use of an anti-PD-1 antibody promotes CD8^+^ cytotoxic T cell function and reduces the disease burden in a BRAFV600E^CD11c^ transgenic mouse model. Moreover, the combination of the anti-PD-1 antibody with MEK inhibitors resulted in a synergistic effect (62).

Targeting of senescent LCH cells and the characteristic inflammatory microenvironment in the lesions represents another potentially promising therapy option for LCH. Senescent LCH cells upregulate proteins of the BCL-2 family (i.e. BCL-2, and BCL-XL) that promote persistence and confer resistance to treatment. Treatment of *BRAF^V600E^* bone marrow-derived DCs with the BCL-2 family inhibitor ABT-263 (navitoclax) induced apoptosis (68) suggesting that inhibitors targeting the anti-apoptotic BCL-2 family might be of therapeutic interest in LCH. The SASP is a strong contributor to the detrimental inflammatory microenvironment in the lesions and targeting SASP has been suggested as a therapeutic option. Studies have provided evidence that SASP is promoted by the mammalian target of rapamycin (mTOR) pathway (119). Targeting SASP by treatment with the mTOR inhibitor rapamycin achieved reduction of the disease burden in *in vivo* pre-clinical models (51). Moreover, inhibition of the key SASP cytokine TNFα with TNFα inhibitors (i.e. infliximab, etanercept) has shown to provide benefit in pre-clinical models (52) but had varying results in the treatment of patients with LCH (120–122).

## Conclusion

To conclude, an increasing number of studies have focused on unraveling the molecular mechanisms that drive the pathological progression of LCH. The discovery of recurrent activating mutations of the MAPK pathway was a major breakthrough in understanding the disease pathobiology and led to the development of novel *BRAF^V600E^*-driven murine and human models of LCH. Due to the better understanding of the disease mechanisms and the continuous effort of multi-national trials, the outcomes of patients with severe multi-system LCH have dramatically improved over the years. Although targeted therapy with BRAF and MEK inhibitors has been shown to be effective at temporarily controlling the disease, the high relapse rates following cessation of the treatment is a major drawback of this treatment strategy. Therefore, the identification of novel therapeutic targets and the development of innovative treatment concepts is one of the main goals currently being tackled by the scientific community. One such interesting concept includes the targeting of OIS and SASP in LCH cells. Promising positive results in murine and humanized pre-clinical models might be quickly translated into therapeutic concepts and help in the treatment of the disease.

Although the understanding of LCH has exponentially increased and novel possible targets have been identified, this represents only the tip of the iceberg in understanding this complex disease. Further studies aiming to better understand the pathobiology of LCH will likely contribute to the enlargement of treatment options and improvement of treatment efficacies.

## Author contributions

TS: Conceptualization, Visualization, Writing – original draft, Writing – review & editing. JF: Visualization, Writing – review & editing. GS: Funding acquisition, Supervision, Validation, Writing – review & editing. AR: Funding acquisition, Supervision, Validation, Writing – review & editing.
